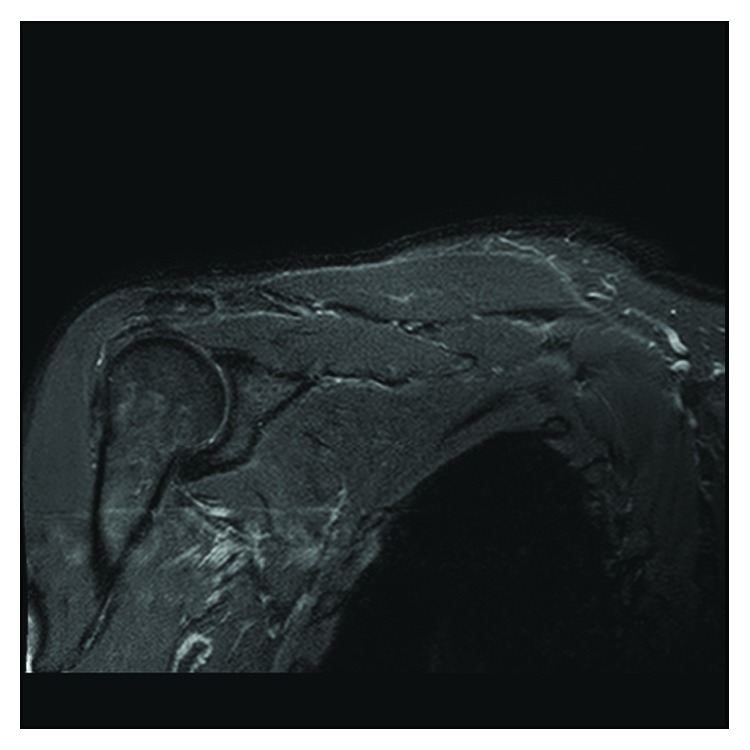# Hyperbaric Oxygen as an Adjunctive Therapy Postfasciotomy for Unilateral Supraspinatus Rhabdomyolysis and Compartment Syndrome

**DOI:** 10.1155/2018/5485767

**Published:** 2018-12-30

**Authors:** G. Hoy, C. Hasenkam, A. Fock, C. McLean

**Affiliations:** ^1^Melbourne Orthopaedic Group, Monash University, Melbourne, VIC, Australia; ^2^University of Melbourne, Melbourne, VIC, Australia; ^3^Intensive Care and Hyperbaric Medicine Unit, Alfred Health, VIC, Australia; ^4^Department of Medicine CCS, Monash University, Melbourne, VIC, Australia

## Abstract

We present a case of severe supraspinatus muscle rhabdomyolysis following overexertion in a young male. Preexisting risk factors included illicit drug use. Even single muscle rhabdomyolysis can cause significant renal failure, and in our case the use of intravenous flushing was used in conjunction with hyperbaric oxygen after muscle compartment fasciotomy to maximize muscle recovery and renal protection in a manual worker (musician). Clinicians should be alert to severe muscle pain requiring narcotics after strenuous use.

## 1. Case Report

A 34-year-old male, right-hand-dominant professional musician presented with a 48-hour history of severe right shoulder pain of sudden onset following a new weights regime. Initially feeling tight, the right shoulder pain was progressive, interrupted sleep, and was refractory to analgesics. Endone was prescribed at initial presentation to the emergency department 24 hours earlier, following a provisional diagnosis of muscle strain. The patient reported no urinary symptoms and had no significant previous medical history, apart from intermittent recreational cocaine use. On examination, global right shoulder weakness and pain on left lateral cervical flexion was apparent, suggesting a possible compartment syndrome.

**Figure 1:**
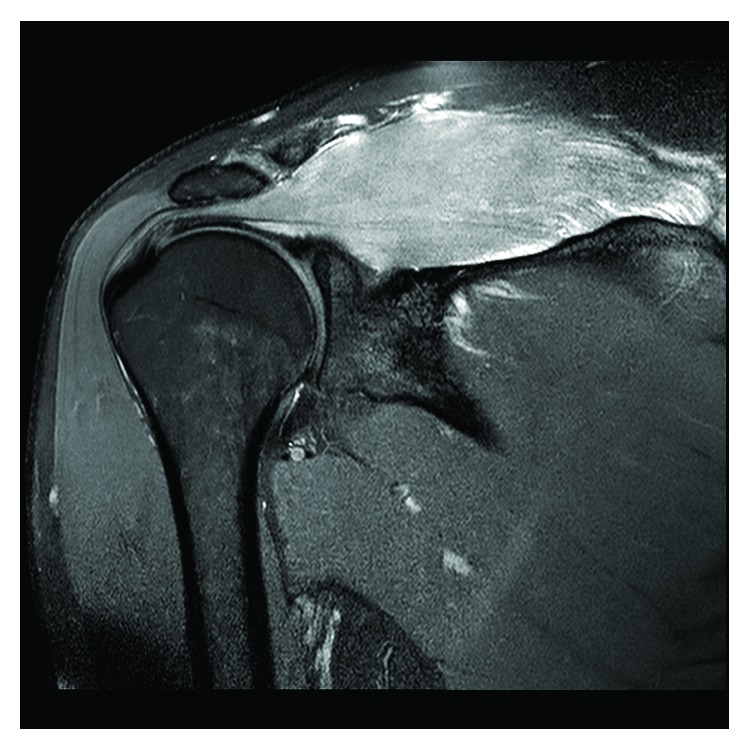


MRI (Figure 1) revealed extensive intramuscular signal change suggesting oedema, denervation, and/or tissue damage, with the supraspinatus being the only muscle affected. The severity of the symptoms warranted emergency decompression, at which time a fasciotomy was performed, poorly contracting muscle was noted, and biopsies were taken.

Laboratory investigations revealed normal renal function and normal electrolyte concentrations. All aspects of the full blood examination were within normal limits. Inflammatory markers were normal, with a C-reactive protein of 2 mg/L (normal 0–10) and erythrocyte sedimentation rate of 4 mm/hr (normal < 15). Creatine kinase (CK) was the only abnormal finding, with a peak level of 17,223 U/L (normal 0–240) at presentation and a level of 13,148 U/L five hours later (immediately prior to fasciotomy).

Emergency right supraspinatus compartment fasciotomy was performed and necrotic tissue debrided. Muscle biopsy results demonstrated skeletal muscle fibre rhabdomyolysis with intervening oedema. Scattered clusters of degranulating perivascular eosinophils were also noted, in the absence of other inflammatory infiltrates.

Postsurgical management involved renal “flushing” with high-flow intravenous supplementation over 72 hours in a high-dependency step-down unit. Renal function monitoring via regular testing was instituted until the CK level dropped below 5000 U/L.

Secondary referral was made to a hyperbaric unit for consideration of hyperbaric oxygen therapy (HBOT). The patient completed seven treatments of HBOT over a five-day period, with three treatments within 24 hours post fasciotomy. The patient was discharged with no pain, normal renal function, and a decreasing CK level.

One month later, the patient had a full active range of motion of the right shoulder in the absence of painful symptoms. MRI review at 6 months post fasciotomy (Figure 2) revealed a largely normal right supraspinatus and rotator cuff, with complete resolution of changes seen on previous MRI.

## 2. Discussion

The most common causes of rhabdomyolysis include direct muscle trauma, overexertion [1], immobilisation, and pharmacologic agents [2]. Overexertion is the most likely precipitant in the current case, with onset of painful symptoms following a weights-based training session completing unaccustomed exercises at increased resistance.

Muscle biopsy taken at fasciotomy suggests a possible mixed aetiology, with perivascular degranulating eosinophils found in the absence of other inflammatory infiltrates. This pattern has been linked with illicit drug use, including cocaine [3]. Rhabdomyolysis from cocaine abuse has been widely reported [3–8]. Although the mechanism is not completely understood, the combination of cocaine-induced local vasoconstriction and direct myocyte toxicity is believed to be involved. The possible mixed aetiology of both overexertion and cocaine use may partly explain the unusually focal unilateral supraspinatus rhabdomyolysis in this case.

Many complications are associated with rhabdomyolysis. Acute renal failure is the most common, occurring directly as a result of myoglobin toxicity to the glomerulus. Due to the minimal skeletal muscle mass of the supraspinatus, systemic effects such as acute renal failure and urine changes were not present and there were also no electrolyte abnormalities. Despite there being no systemic complications, local skeletal muscle compartment syndrome (SMCS) developed.

SMCS is a medical emergency, and it occurs when intracompartment pressure exceeds capillary perfusion pressure, resulting in hypoxia and ischaemia of the supplied tissues [9]. Following injury or trauma, oedema or bleeding within the confines of a muscle compartment increases the pressure within that compartment. When the increased pressure collapses the microcirculation, hypoxia and ischaemia follow, leading to a self-perpetuating cycle of oedema leading to tissue ischaemia and vice versa [9]. In this case, the dense fascial compartment enclosing the supraspinatus, in combination with the rigid bony confines of the scapula inferiorly, lead to the development of increasing pressures and SMCS [10, 11]. Early detection of SMCS is critical to maximising salvaged tissue and following diagnosis, with urgent fasciotomy indicated.

HBOT refers to the breathing of 100% oxygen under increased atmospheric pressure, and numerous reports have identified its benefit in rhabdomyolysis and SMCS [12–15] The Undersea and Hyperbaric Medical Society (UHMS) have approved the use of HBOT as an adjunctive therapy postfasciotomy in the following situations: continuing oedema or ischaemic tissue; unclear demarcation between viable and nonviable tissue; residual neuropathy; or prolonged ischaemia time [9]. 1–2 daily HBOT treatments are indicated for up to 7–10 days post fasciotomy, with the resolution of symptoms and complications guiding the decision to cease HBOT [9].

Through minimising both hallmark features of SMCS, hypoxia and oedema, HBOT has a number of benefits postfasciotomy and in rhabdomyolysis and compartment syndromes generally. HBOT boosts arterial oxygen partial pressure, directly increasing oxygen supply to hypoxic tissue, as well as minimising cellular metabolic dysfunction. This also helps stimulate angiogenesis and activate fibroblasts and macrophages to aid wound healing. By minimising endothelial dysfunction, HBOT reduces reactive oxygen species production, reducing their direct insult to local tissue.

Lastly, hyperoxygenation achieved through HBOT induces vasoconstriction, reducing blood flow to damaged tissues by up to 20% as well as producing a direct osmotic effect, reducing local oedema whilst maintaining oxygen delivery [12, 16]. Numerous reports and studies involving animal models have documented the benefits of HBOT postfasciotomy, and as in this case, HBOT should be utilised where available to maximise salvaged tissue, wound healing, and overall outcomes. If possible, and depending on the severity of the SMCS and the exclusion of other conditions, HBOT may be considered *before* fasciotomy, as it may prevent the need for surgery altogether.

### 2.1. Lessons from Practice

- Specific personal training fitness regimes are an increasing exercise modality in the community.
- Illicit drug use is an important aspect of every medical history.
- Both illicit drug use and high-intensity fitness regimes are important potential causes of rhabdomyolysis.
- Prompt diagnosis of rhabdomyolysis is critical to preventing complications such as acute renal failure and compartment syndrome.
- Hyperbaric oxygen therapy is an excellent adjunctive treatment postfasciotomy.

## Figures and Tables

**Figure 2:**